# The lateral prefrontal cortex of primates encodes stimulus colors and their behavioral relevance during a match-to-sample task

**DOI:** 10.1038/s41598-020-61171-3

**Published:** 2020-03-06

**Authors:** Philipp Schwedhelm, Daniel Baldauf, Stefan Treue

**Affiliations:** 1 Cognitive Neuroscience Laboratory, German Primate Center, Kellnerweg 4, 37077 Goettingen, Germany; 2 Center for Mind and Brain Sciences, University of Trento, Via delle Regole 101, 38123 Mattarello, TN Italy; 3 Functional Imaging Laboratory, German Primate Center, Kellnerweg 4, 37077 Goettingen, Germany; 4 Institute of Molecular and Clinical Ophthalmology Basel, Mittlere Strasse 91, 4031 Basel, Switzerland; 5 Bernstein Center for Computational Neuroscience, Am Fassberg 17, 37077 Goettingen, Germany; 6 Faculty of Biology and Psychology, University of Goettingen, Gosslerstraße 14, 37073 Goettingen, Germany; 7 Leibniz ScienceCampus Primate Cognition, German Primate Center, Kellnerweg 4, 37077 Goettingen, Germany

**Keywords:** Attention, Cognitive control, Decision

## Abstract

The lateral prefrontal cortex of primates (lPFC) plays a central role in complex cognitive behavior, in decision-making as well as in guiding top-down attention. However, how and where in lPFC such behaviorally relevant signals are computed is poorly understood. We analyzed neural recordings from chronic microelectrode arrays implanted in lPFC region 8Av/45 of two rhesus macaques. The animals performed a feature match-to-sample task requiring them to match both motion and color information in a test stimulus. This task allowed to separate the encoding of stimulus motion and color from their current behavioral relevance on a trial-by-trial basis. We found that upcoming motor behavior can be robustly predicted from lPFC activity. In addition, we show that 8Av/45 encodes the color of a visual stimulus, regardless of its behavioral relevance. Most notably, whether a color matches the searched-for color can be decoded independent of a trial’s motor outcome and while subjects detect unique feature conjunctions of color and motion. Thus, macaque area 8Av/45 computes, among other task-relevant information, the behavioral relevance of visual color features. Such a signal is most critical for both the selection of responses as well as the deployment of top-down modulatory signals, like feature-based attention.

## Introduction

Recent electrophysiological studies indicate that task-relevant visual information are encoded by the lateral prefrontal cortex (lPFC)^1–3^. In addition to such sensory information, the memory of searched-for visual features and current task rules are also encoded by lPFC neurons^4–13^. Another line of research supports the view that the lPFC of macaques is involved in the preparation of an attentional signal^9,14–16^. This signal is thought to be relayed to visual cortex, where it enhances the processing of behaviorally relevant information^17–23^, which ultimately leads to behavioral advantages, e.g. higher task accuracy and/or faster reaction times for attended as compared to unattended stimuli^24^.

Such a modulatory influence on the neural representation of behaviorally relevant stimuli and their visual features requires a rapid encoding of visual information in lPFC along with an immediate selection of relevant features and their communication to visual areas, both of which take place in lPFC^25–27^.

Thus, the prerequisites for the computation of modulatory, cognitive signals are in place and in fact, the Frontal Eye Fields (FEF), a core node of the saccade generation network within lPFC^28–30^, have been identified as central elements of the attentional network. Sub-threshold microstimulation of FEF causes subtle behavioral effects^31,32^ and changes in the responses of visual cortical neurons that resemble the modulatory effects of spatial attention^33–35^. Also within lPFC, the functionally defined ventral prearcuate region (VPA^15^) has recently been suggested as a likely source of signals guiding the allocation of feature-based attention.

In this report we investigate the activity of a prefrontal region directly anterior to the FEF of the macaque monkey, within the rostral part of area 8A^36,37^. This patch of cortex (here termed 8Av/45^38^ lies in between FEF and VPA and is heavily interconnected with both visual cortical areas and also within lPFC^39–42^, rendering it an ideal candidate to receive ‘bottom-up’ visual input and communicate it to other prefrontal structures. Another likely function of 8Av/45 would be to relay ‘top-down’ modulatory signals to visual cortex, or among prefrontal areas, like FEF and VPA. Thus, the neuronal computations that are performed at the level of 8Av/45 may be related to stimulus perception, motor-planning or the computation of higher cognitive signals, like feature-based attention and decision-making.

In this report, we analyze neuronal data recorded while monkeys performed a delayed feature-match-to-sample task. In a decision-theoretic model^43^ and throughout a single trial of such a task, animals need to perceive the sample stimulus, memorize it, perceive the test, compare it to the sample and finally formulate and execute a motor plan. To this end we show that 8Av/45 robustly encodes stimulus colors both during sample and test stimulus presentations. Further, the trial-by-trial behavior of the monkeys can be predicted, well before motor execution and especially for behavior that is based on stimulus color. This suggests that for task conditions in which a decision can be made solely based on the stimulus’ color, 8Av/45 activity may be causally linked to the monkey’s behavior.

We further tested a more complex conjunction-matching task, which required the monkeys to integrate color and motion features of the stimuli. Here we could separately analyze matching colors and motion directions and found that the activity of 8Av/45 is informative about the behavioral *relevance* of a current stimulus’ color, but not its motion direction. This signal appears in addition to the robust encoding of color *identity* and can therefore influence the selection or execution of movement plans. Importantly though, the ‘color-match’ signal appears independent of motor execution and may therefore also be used by other prefrontal structures to generate modulatory signals like attention or in the updating of decision-related variables and the preparation or competition of actions.

## Results

**Figure 1:**
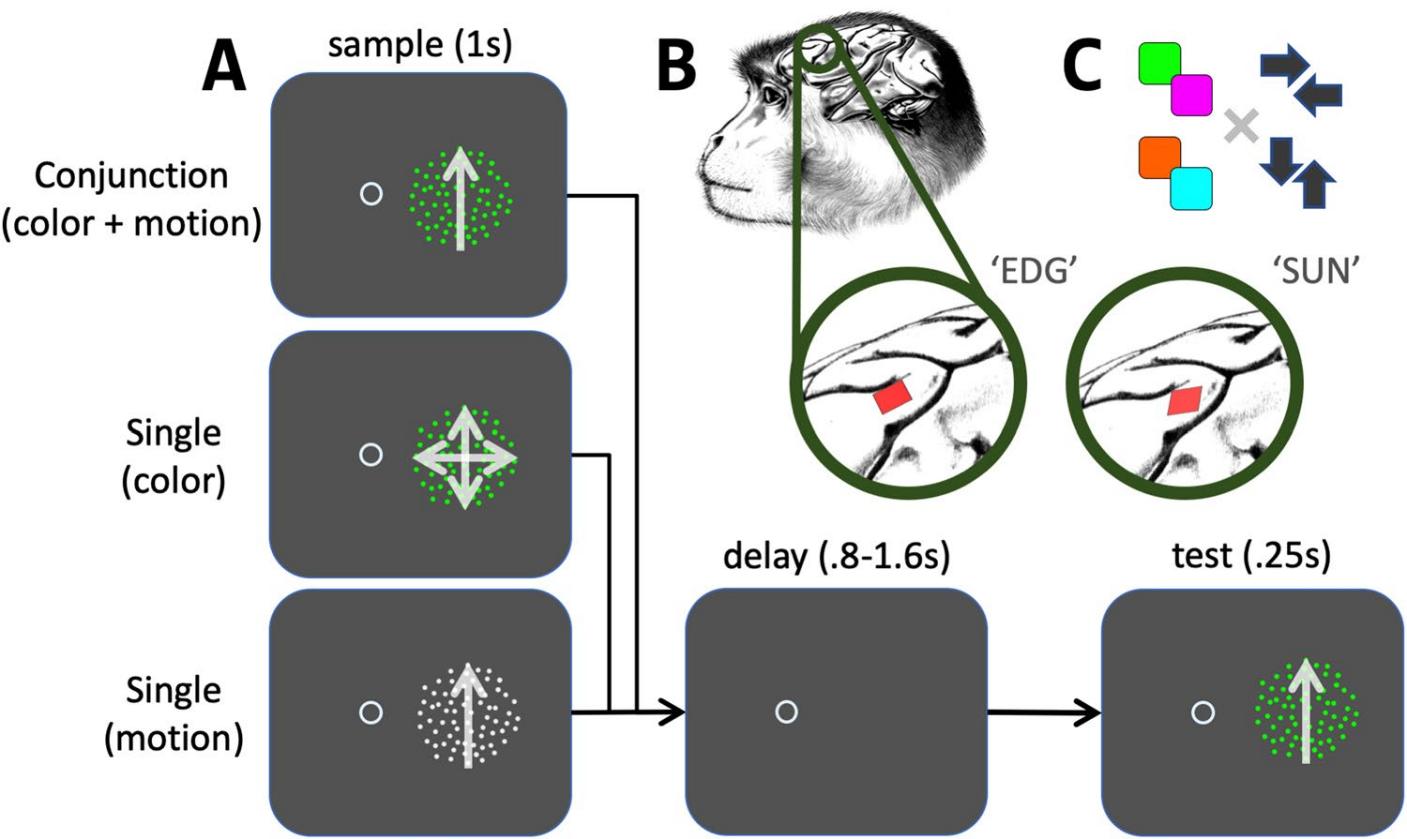
A delayed match-to-feature task for monkeys. (**A**) The animals started an individual trial by fixating the central fixation point and releasing a manual push-button. Next, a sample was presented for 1 second. The sample cued the monkeys as to the trial type (either conjunction-matching, motion-matching or color-matching). Simultaneously, the sample stimulus contained the relevant visual feature(s) (color and/or motion) that had to be remembered and then matched to the test stimulus. Stochastic motion or a grey color signaled that the respective feature was irrelevant for the current trial. After a variable delay (800–1600 ms) we presented a test stimulus for 250 ms. The test always moved coherently in a cardinal direction and was always colored in one out of four isoluminant colors. The animals responded by depressing the response button when the test stimulus matched the feature(s) of the sample and received a liquid reward upon correct responses. If the test did not match the sample, the animals were rewarded for not responding within 600 ms of test stimulus onset. Eye fixation had to be maintained throughout sample, delay and test epochs. (**B**) We recorded local field potentials from the animals left lPFC by means of chronically implanted 96-channel microelectrode arrays. For both animals we analyzed the simultaneously recorded data from all available channels. Drawings by Klaus Lamberty, Deutsches Primatenzentrum GmbH. (**C**) We arbitrarily grouped colors and motion directions into pairs of features. This ensured that during test presentation, the likelihood of a target presentation was 50%.

Local field potentials were recorded from region 8Av/45 of the lateral prefrontal cortex of two macaques performing a delayed match-to-sample task. The task required the animals to report the presence of match stimuli and ignore non-matching stimuli. In single feature trials, matches had to be identified based on one stimulus feature of the test (color or motion). In conjunction trials, two features (color and motion) had to be matched to the sample stimulus. The animals reported matching stimuli by depressing a manual push-button and had to withhold such a response for non-matches. They received a liquid reward for both types of correct responses to the test stimulus. The three task conditions were randomly interleaved and the featural properties of the sample stimulus cued the animals about the task type of a trial (Fig. 1A).

### Animals used both target features for conjunction matching

We first analyzed the behavioral performance of both animals separately for each of the possible test stimulus configurations in each behavioral task. In the conjunction-match task, the test stimulus could mismatch the sample features in both motion and color, or only in a single feature, allowing for an in-depth analysis of the behavioral strategy of the monkeys. For this purpose, we grouped the mismatch trials of the conjunction task according to their three types (i.e. color-, motion- and complete-mismatch) and then separately contrasted them with match trials to calculate sensitivity indices d’. This allows to estimate how much each visual feature contributes to the behavioral performance of the monkeys during conjunction match trials.

**Figure 2:**
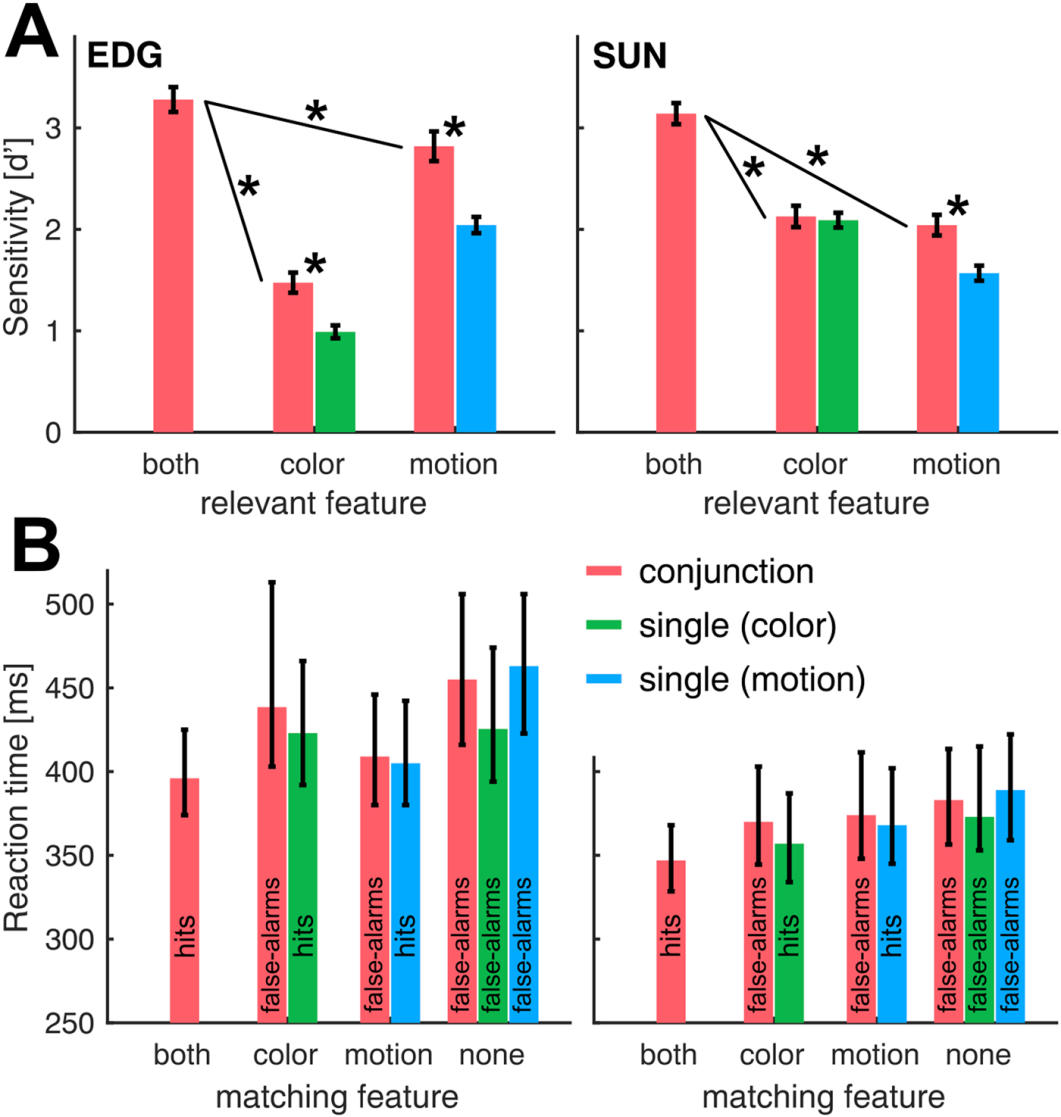
Both color and motion features were relevant for the monkey’s behavior. (**A**) Sensitivity indices d’ were calculated separately for each task-relevant stimulus feature. In conjunction trials, we contrasted the responses to target stimuli matching both sample features (color and motion) with responses to non-target stimuli matching either none, or only a single feature. We compared those values with the sensitivity in single task trials, in which only one visual feature was behaviorally relevant. Error bars represent standard errors across n = 16 and n = 25 sessions, for monkey EDG and SUN, respectively. Stars indicate significant differences. (**B**) Median reaction times plotted separately for stimuli matching both searched-for features (hits in conjunction trials), for stimuli matching either color or motion, or stimuli matching none of the sample features (false-alarms). Those data are plotted as a function of matching features and thus contain hit trials and false-alarms, as indicated by the bar labels. Error bars indicate first to third quartile ranges.

As expected, we found that the monkeys’ sensitivity to distinguish stimuli was significantly lower when only one of the visual features composed the difference between match and non-match stimuli. This effect was present for both monkeys and for both features (N = 16 and N = 25 sessions for monkey EDG and SUN respectively; motion: p = 1.6e-5 and p = 2.4e-15; color: p = 3.4e-15 and p = 5.1e-16, all paired-sample t-tests; Fig. 2A). Monkey EDG showed a bias towards using the motion information for his decision, as indicated by a significantly higher d’ for trials in which only the motion was relevant, as compared to the color information (p = 7.3e-10, paired-sample t-test, N = 16 sessions). Monkey SUN, on the other hand, did not show such a bias (p = 0.23, paired-sample t-test for color- and motion-relevant conjunction trial performance, N = 25 sessions; Fig. 2A).

We also contrasted conjunction task performance with the performance in interleaved single-task trials, in which only one feature (color or motion) was present in the sample. In those trials, the sample stimulus either had a grey color, cueing the monkeys to ignore color and match only the motion of the sample to the test (motion task), or the sample stimulus moved incoherently in random directions, cueing the monkeys to ignore motion of the test and match only the color of the sample to the test (color task). Critically, the test stimulus always moved in one of four directions and had one of four colors (Fig. 1A). Thus, the monkeys had to ignore the task-irrelevant feature of the test and base their decision only on the relevant feature (Fig. 2A).

The monkeys’ behavioral performance in the two single-feature tasks was similar, albeit slightly lower than in conjunction trials with just one feature available (p = 0.001 and p = 0.77 for color vs. color-control trials and monkeys EDG and SUN, respectively; and p = 2.8e-5 and p = 1.7e-6 for motion vs. motion-control trials, all paired-sample t-tests, N = 16 and N = 25 sessions for EDG and SUN, respectively).

Figure 2B shows the reaction times for trials in which the monkeys reported a test stimulus as a match (i.e. hit trials and false-alarms). We normalized reaction times to speed by calculating the reciprocal of individual latencies and then tested with two-sample t-tests for differences between groups. For the conjunction task, we find that false-alarms are preceded by significantly longer reaction times than hit trials for both animals and all comparisons between hit trials and any type of false-alarms (for EDG: N = 1546 hit trials vs. 597 motion-matching (p = 1.2e-7), 60 color-matching (p = 3.4e-13) and 10 neither-matching false-alarms (p = 3.9e-4); for SUN: N = 2404 hit trials vs. 228 motion-matching (p = 1.7e-26), 268 color-matching (p = 5.8e-21) and 12 neither-matching false-alarms (p = 0.002); all two-sample t-tests; see Fig. 2B). Except for the color control, this was also the case for the single feature tasks (EDG: p = 0.36 and p = 2.6e-9 for color and motion tasks, N = 781 motion-task hits, 23 false-alarms, N = 802 color-task hits, 298 false-alarms; SUN: p = 1.5e-6 and p = 3.6e-6 for color and motion task, N = 1210 motion-task hits, 181 false-alarms, N = 1212 color-task hits, 107 false-alarms; all two-sample t-tests). Further, both animals showed significant reaction time differences for color and motion task hits (p = 1.1e-9 and p = 9.7e-13, for EDG and SUN, respectively; for N see above), but the sign of this effect was inconsistent across animals, indicating that overall, matching the motion and the color feature imposed a comparable challenge for the animals.

To confirm the robustness of our comparisons, we evaluated the significance of effects based on a Bonferroni-corrected alpha of 0.005. We also repeated all statistical tests using non-transformed reaction times and also using non-parametric Wilcoxon tests. This did not qualitatively change our results. Lastly, we addressed the possibility that the animals learned specific sample-test lookup tables instead of abstract task rules by calculating the behavioral performance separately for each sample composition. Since the differences in behavior for different samples were small, we conclude that both monkeys learned the abstract rules of our delayed match-to-feature task (Fig. 1 and Supplementary Fig. S1).

### 8Av/45 encodes the color features of stimuli

We recorded local field potentials (LFPs) from chronically implanted 96 channel Utah-arrays placed in each animal’s left inferior principal gyrus, just below the posterior end of the principal sulcus (Fig. 1B). After data acquisition, we band-pass filtered the signals from 1–80 Hz, selected only correct trials (i.e. hits and correct rejections) and balanced the dataset regarding all analyzed task-relevant variables (e.g. trial types, outcomes and stimulus configurations) by randomly excluding overrepresented trials. We then performed several decoding analyses by training support-vector machines to separate the neuronal data based on task-related variables (see also Methods section). In the first of those analyses we investigated whether the visual features of the shown stimuli were decodable from 8Av/45 activity.

**Figure 3:**
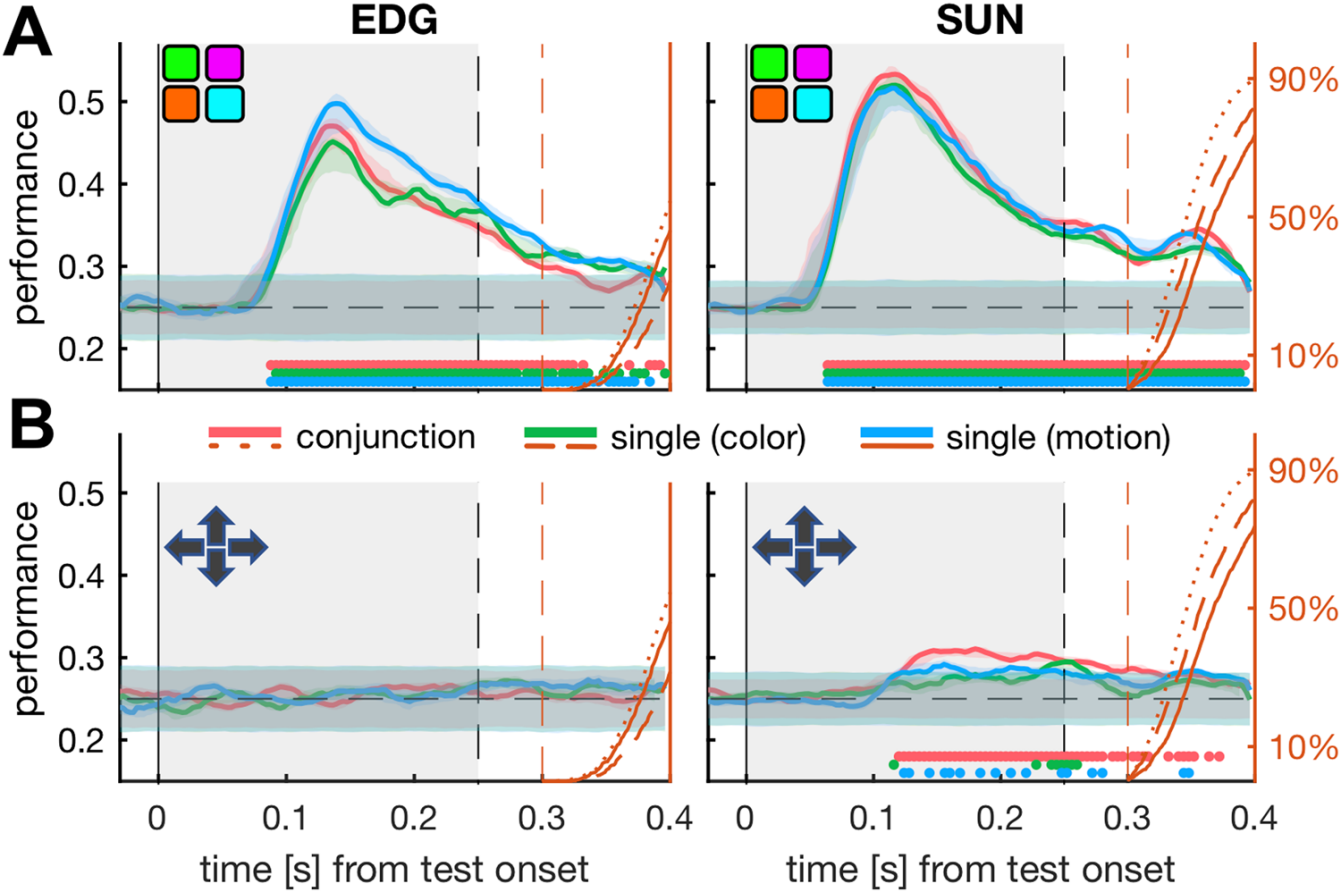
Test color can be decoded from 8Av/45 activity. (**A**) We trained support vector machines to separate four different stimulus colors of the test stimulus based on time-locked 8Av/45 data. We estimated the performances of the classifiers with a 20-fold cross-validation procedure and contrasted the results with distributions of 500 runs of the same data, but with randomly shuffled trial labels. Here, we plot performance as the moving average in 28 ms sliding-windows with corresponding 95% confidence intervals. Chance performances are plotted as 99.9% confidence intervals of the shuffle distributions as overlapping, shaded areas. Above the x-axis, dots in corresponding colors indicate for which time-bins the decoding probability significantly differs from chance, evaluated at a α = 1e-4. The grey, shaded area illustrates the test stimulus duration. Orange, right-hand axes correspond to cumulative reaction-time distributions for each trial group and their corresponding target trials. Here, 100% equals to all target trials being terminated by the monkey. The orange, dashed line illustrates the fastest possible reaction time (300 ms, as defined by trial inclusion criteria). (**B**) Like A, but for the four cardinal motion directions.

We found that during test stimulus presentation, 8Av/45 activity is highly informative about the color of the stimulus (see Fig. 3A). To estimate the significance of this decoding, we determined the chance performance of our decoders by randomly shuffling the trial labels before training and repeating this procedure 500 times. We contrasted the resulting noise distribution with the unpermuted data using z-tests and defined a threshold of p < 1e-4 as significant decoding performance. Finally, we defined the center of the earliest 40 ms window in which all time-points yielded a significant performance as the onset of decoding.

For both monkeys, the activity of 8Av/45 became informative about the stimulus color shortly after test onset. The onset latencies for this decoding were as short as 84 ms following stimulus onset (monkey SUN, all tasks) and starting from 108 ms (motion and conjunction tasks), and 112 ms (color task) for monkey EDG. For statistical testing, we estimated onsets for all 20 testing folds of each classifier and then compared the resulting distributions between the three task types. For both monkeys, the onset latencies for color decoding were not significantly different between tasks (EDG: p = 0.059, p = 0.34 and p = 0.48; SUN: p = 0.24, p = 0.02 and p = 0.25, for color vs conjunction, color vs motion and motion vs conjunction tasks, respectively; all two-sample t-tests with Bonferroni corrected α = 0.0167, N = 20 testing folds).

We also tested whether the decoding performance for color features differed significantly between the three task conditions. For this analysis, we compared the predictions of each group of 20 testing folds with two-sample t-tests, evaluated at a Bonferroni corrected α = 0.0167. As before, we searched for epochs of significant differences that were at least 40 ms long, but found that for no task-type and monkey such epochs existed (see also Supplementary Fig. S2). In other words, whether color, motion or both features were relevant for the monkey’s behavior did not change the strength of color encoding in 8Av/45.

Confirming this observation, we obtained a similar pattern of results for sample stimulus presentations, for which we also performed the same decoding analysis (see Supplementary Fig. S3). Therefore, the robust and low-latency decodability of color information during both sample and test presentations likely constitutes a fast, bottom-up visual input to 8Av/45.

In contrast to our success in decoding of stimulus color, we were unable to reliably decode the stimulus motion from 8Av/45 activity. Only from monkey SUN, and only during conjunction trials, motion could be decoded, starting at 140 ms after stimulus onset (Fig. 3B). This was the case for the test stimulus, and also for the sample stimulus (see Supplementary Fig. S3). We also tested whether any information about sample features would extend into the memory period but found that even before the end of the presentation of the sample, the decoding performance for both color and motion returned to baseline. We also did not observe a recall of sample information before test stimulus onset (see Supplementary Fig. S4). Lastly, we investigated whether test color and test motion could be estimated from single channels of the chronic electrode arrays. Here we found that for both monkeys, the decoding results obtained from single channels mimicked the results obtained by combined decoding from all channels. Further, for both monkeys, recording sites that were maximally informative about the stimulus color tended to be clustered together (see Supplementary Fig. S5 and Video S6).

### Trial-by-trial behavior can be predicted from 8Av/45 activity

We next investigated whether the behavioral outcome of successful trials (i.e. hit trials and correct rejections) was predictable from 8Av/45 recordings. For this purpose, we first pooled trials with stimuli of all features and then trained support-vector machines to separate the neuronal data based on the presence or absence of trial-terminating button-presses (see also Methods section). Importantly, before performing this decoding analysis, we again balanced the dataset such that no type of trial outcome and no stimulus identity (motion or color) was overrepresented. Thus, the previously shown color selectivity of 8Av/45 cannot explain significant decoding performances, because the trial classes (in this case: hits and false-alarms) contain equal numbers of trials of each stimulus color, motion or any combination thereof.

**Figure 4:**
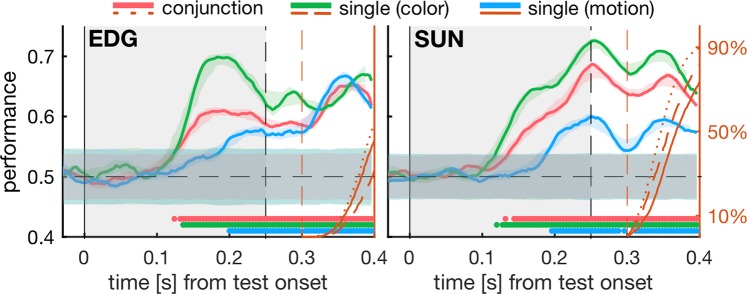
Trial-by-trial behavior can be predicted from 8Av/45 activity. We trained support vector machines to separate correct trials with button presses (hit trials) from trials without button presses (correct rejections) based on time-locked LFPs recorded from 8Av/45 during test stimulus presentations. When the monkeys’ behavior was based only on stimulus color or a conjunction of color and motion (conjunction task), this decoding became significant between 148–164 ms after test stimulus onset for both monkeys. For decisions based only on stimulus motion, we observed lower decoding performances and later onsets (216–220 ms). Orange, right-hand axes correspond to cumulative reaction-time distributions for each trial group and their corresponding target trials. Dashed, orange lines at 300 ms indicate the start of response windows. No button presses occurred before this time. Other analysis details and plot layout like in Fig. 3.

We found that for both monkeys, their upcoming behavior could be reliably predicted from 8Av/45 activity well before the motor response took place (see Fig. 4). For single-feature trials, in which behavior had to be based on the color feature alone (color task), this prediction became significant already at 156 ms and 148 ms following test stimulus onset (for monkey EDG and SUN, respectively; center of 40 ms wide epochs with only significant decoding). In contrast, median reaction-times during the color task were 423 ms and 357 ms following test onset, for EDG and SUN, respectively (Fig. 2B and Fig. 4, orange axes). Additionally, trials with reaction times shorter than 300 ms were excluded from analysis (see also Methods section), such that significant decoding of task behavior was present at least 144 ms before motor execution.

During the conjunction task, when the monkey’s behavior based on both color and motion features, the classification performance also became significant 152 ms and 164 ms after test stimulus onset (Fig. 4). Behavior which based only on the stimulus motion (motion task) could be decoded with low performance and long latencies (220 ms and 216 ms, for monkey EDG and SUN, respectively). We used the performance of individual testing folds to test for differences across task conditions, with the procedure described previously. Here we found that for both monkeys the performances of color and conjunction tasks had significantly different onsets (EDG: p = 1.9e-4 and SUN: p = 6.1e-3, two-sample t-tests, N = 20 folds), while we could not perform the same analysis for the motion task, because not all testing folds had a 40 ms wide epoch with significant decoding performance. Between task conditions, we found that decoding performances became significantly different (Bonferroni corrected α = 0.0167) between color and conjunction trials after 168 ms for EDG and 216 ms for SUN. Color and motion trials were significantly different already after 152 ms for both monkeys. Between motion and conjunction trials, the difference reached significance after 152 ms for monkey SUN, but did not become significant for monkey EDG (see also Supplementary Fig. S7).

This pattern of results was not reflected in the recorded task behavior of the monkeys (see Fig. 4, orange axes for a cumulative distribution of reaction times). In fact, at least for monkey EDG, median response latencies were shorter for motion than for color trials (2-sample t-test, p = 1.1e-9, N = 802 color task hits, N = 781 motion task hits; see Fig. 2B) and the behavioral performance was higher during the motion task than during color task (paired-sample t-test, p = 3.8e-7, N = 16 sessions; see Fig. 2A).

Lastly, we again tested how well single channel LFPs recorded from the prefrontal arrays could be used to predict trial behavior. We found that for both monkeys, very few channels were informative early (i.e. 100–200 ms) during test stimulus presentation, and that those channels tended to form clusters of electrodes, that were spatially separated from informative electrodes during color decoding. At time-points closer to the monkeys’ responses, however, single-channel decoding performance of trial outcomes tended to be more homogeneously spread across the prefrontal arrays (see Supplementary Fig. S8 and Video S9).

### The behavioral relevance of colors can be read out from 8Av/45 even in the absence of responses

In order to investigate whether 8Av/45 activity represents a given trial’s motor preparation, or other, more categorical (e.g. decision-related) variables, we next analyzed only trials in which the behavioral outcome was the same across trials, but the stimulus feature that determined the trial-by-trial decision was known. This distinction could be made by analyzing those trials of the conjunction task, where test stimuli matched only one of the two searched-for features, but not the other. In other words, we contrasted non-target stimuli that did not match to any of the sample features with non-target stimuli that matched either the color or the motion feature, but not both.

**Figure 5:**
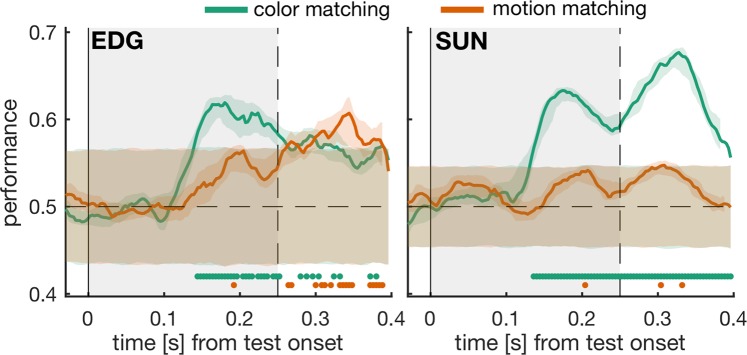
The behavioral relevance of stimulus colors can be read out from 8Av/45 activity. For conjunction matching trials, we contrasted presentations of correctly ignored test stimuli that mismatched the sample in color and motion with test stimuli that were also correctly ignored but matched either the searched-for color or motion, but not both. We trained support vector classifiers to separate these two types of non-matching test stimuli and plotted the decoding performance over time and against corresponding noise distributions. Significant time-bins are indicated by color-corresponding dots above the x-axis. Other analysis details and plot layout like in Fig. 3.

For both groups of test stimuli, we only analyzed trials in which the monkeys correctly rejected the stimuli as non-matching. Again, we balanced the dataset regarding stimulus identity (color and motion features) and trial outcome and then trained our classifiers to distinguish between these half-matching test stimuli and complete mismatching tests. As opposed to decoding the behavioral (motor) outcome, this analysis detects whether 8Av/45 activity is informative about a match of the test to the searched-for feature in either the color (Fig. 5, green traces), or the motion (Fig. 5, orange traces).

We found that 8Av/45 activity can be used to read out whether a test stimulus matches the searched-for color (see Fig. 5). The latency of this read-out (164 ms and 156 ms in monkey EDG and SUN, respectively) is comparable to the prediction latency for trial behavior in the combined task (Fig. 4). In this case, however, a pre-motor signal is unlikely to be the cause of the significant decoding, since all analyzed trials resulted in the same motor outcome.

Whether the motion of a test stimulus matches the motion of a previously shown sample could also be read out from 8Av/45 activity, but only for monkey EDG and mainly at latencies that fall within the monkeys’ typical reaction time distributions (see also Fig. 4, orange axes). Although we observed rising decoding performances for motion matches in both monkeys, these trends never reached significance for any 40ms-wide epoch, in both monkeys. The onset of significant color match encoding was similar across monkeys (i.e. not significantly different; p = 0.17, two-sample t-test, each N = 20 testing folds), while in monkey EDG, the difference between color matching and motion matching performance narrowly misses significance (no 40 ms wide epoch with all p < 0.0167, see Supplementary Fig. S10). For monkey SUN, on the other hand, color and motion match performances are significantly different (onset at 152 ms, Figs. S10 and 5, right panel).

On the level of single channels, it should be noted that especially when comparing color and conjunction tasks, LFP signals from electrodes that were predictive of the monkeys’ task behavior during the color task can also be used to decode color matches of test stimuli in conjunction task trials (see Supplementary Fig. S11 and Video S12). However, an in-depth analysis of single sites of our electrode arrays addressing the presumed functional heterogeneity of different recording locations is outside the scope of this study.

In summary, we show here that 8Av/45 activity can not only be used to decode the color of visual stimuli and predict the behavioral outcome (i.e. motor response) of single trials, but it can also be used to read out the behavioral *relevance* of a presented stimulus color, even in the absence of behavioral responses.

## Discussion

In this study we assessed whether the neuronal activity of lPFC is related to stimulus perception, motor-planning, or higher cognitive signals, like the detection of featural matches during a match-to-sample task. To this end, we recorded local field potentials from chronically implanted microelectrode arrays positioned in two rhesus macaques’ left 8Av/45. The animals performed a delayed visual feature-matching task which required push-button responses once a test stimulus matched a previously presented sample stimulus in its color, motion or a conjunction of color and motion. We found that during stimulus presentation, the color features of samples and test stimuli can be readily decoded from simultaneous recordings of all available electrodes (up to 96 channels). Further, trial-by-trial behavior based on the color features of stimuli could be predicted as early as 148 ms after test stimulus onset. Lastly, in addition to color *identity*, it was possible to decode whether a color was *relevant* for an upcoming decision, even if the behavioral response of the monkeys was the same in all trials.

The activity of lPFC has recently been linked to high-level cortical functions during the preparation and execution of a wide variety of tasks^44,45^, but attributing specific functions to small prefrontal areas is a notoriously difficult task, especially in light of the fact that flexible sensorimotor decisions require parallel computations between different cortical stages^46^. Here however, we demonstrate a specialization of a small patch within lPFC for task-related evaluations of visual color features.

This is partly in conflict with previous findings demonstrating motion selectivity across 8Av/45^1,2^, but could be a result of our tightly constrained recording locations, that covered only an area of 4 mm^2^ in each monkey. We also recorded from a constant depth of 1.5 mm below the cortical surface and therefore did not cover all cortical layers. For our analysis, we pooled data across all recording sites and then determined the linear separability of task-related information with a decoding approach. This analysis technique uncovers informational content that might not be evident from spiking rate data of single units, if analyzed in isolation.

However, while our study is not the first that shows a preference of lPFC for color features over motion features^47^, we show here that even in cases in which color was of no behavioral relevance for the task at hand, 8Av/45 robustly encodes this information. Taken together with our finding that color encoding emerged very rapidly after stimulus onset, we conclude that 8Av/45 likely receives direct ‘bottom-up’ visual input, as was also suggested by tracer studies^39,42^ and the finding of coarse retinotopy directly adjacent to 8Av/45^3^.

Rushworth and colleagues showed that when animals perform color matching tasks, lesions of lPFC impair task performance^48^. Yet, how exactly the lPFC contributes to color-matching behavior is still a matter of debate. It was proposed that lPFC computes a signal related to response-inhibition^9,47^, or the change of mind during evidence accumulation^49^, but based on our data from 8Av/45 one could also hypothesize that lesions of lPFC can impair color perception of the animals.

Our results also indicate that 8Av/45 activity represents the upcoming behavior on a trial-by-trial basis, dependent upon the currently relevant visual feature. While our data do not allow to distinguish response-inhibition from facilitation, we show that the behavior-critical information in 8Av/45 constitutes a signal that indicates the presence (or absence) of a relevant color feature. This ‘match’ signal may be part of a decision-making process that transforms visual information into motor actions. However, since we show that 8Av/45 is specialized on detecting matches of color features, but not for motion, we argue that decision-making itself happens elsewhere in the brain and is only later represented in 8Av/45. Still, our data suggest that 8Av/45 activity may be linked to changes in decision-related variables, like the updating of values and the preparation of actions^50,51^.

Lennert and Martinez-Trujillo^9^ investigated the firing-rates of neurons recorded in close vicinity to the cortical area investigated in this report. They find that 8Av/45 (there termed dorsolateral prefrontal cortex) transforms the relative ordinal rank of colors to a spatial signal that may be used to direct attention onto a behaviorally relevant stimulus. More evidence for the idea that lPFC guides such attentional signals informed by color features comes from lesion studies^52^.

In our study, we used a delayed, feature-match-to-sample task. This task did not contain epochs with sensory uncertainty, like in discrimination tasks. Instead, sample features had to be remembered and then compared to a subsequently shown test stimulus. Despite these differences from typical attentional paradigms, a top-down modulation of visual cortex could have helped the animals to detect searched-for stimuli faster and with higher accuracy among non-matches. Yet, in our 8Av/45 data we did not detect either maintenance of sample information during delay epochs or the recall of such information before test stimulus presentation. Therefore, we argue that it is unlikely that 8Av/45 is involved in a direct (and sustained) modulation of visual cortex.

Here it should be noted, that recent neurophysiological evidence suggests that the lPFC can maintain some working memory in an activity-silent manner^53^. In this scenario, one would expect correlated, low-energy activity resulting from temporary connectivity changes among lPFC units^54^. Separating these ‘baseline emission’ states is not something our decoding approach based on LFP data is well-equipped for. It is therefore possible that working memory is encoded by lPFC, but not reflected in persistent activity of single units.

In this report we show that 8Av/45 activity is informative about whether a stimulus matches a searched-for color and that this signal occurs independently of a monkeys’ behavior. To generate such a match-signal without persistent sample memory, 8Av/45 could access priority- or integrated saliency maps for colors^55,56^. Such maps maintain current behavioral goals, for example the featural composition of a sample stimulus that needs to be compared to a test. They are then available for comparison to the current visual input.

The information computed at the level of 8Av/45 may thus be a signal for when a goal state for a specific comparison is reached^57^. The resulting signal (i.e. the ‘match’ in this case) could be transformed into direct effector output, like a saccade or hand movement, be used to inform a secondary decision-making process, or to increase the gain of those neuronal populations in visual cortex that are responsible for sampling the currently relevant color feature. Exactly how such a signal is utilized will depend on the current behavioral demand imposed by the task at hand.

This unifies seemingly conflicting views on the function of lPFC and its submodules. For the conjunction match-to-sample task used in this study, the color-match signal generated by 8Av/45 can be integrated with other match signals by a decision-making process to compute a given trial’s behavior. During another behavioral task, for example a color-discrimination task that requires selective attention, the same signal may be most helpful in deploying processing resources specifically to currently relevant color features of the visual input. Therefore, 8Av/45 activity may be positioned at the intersection between attention and decision processes, with great relevance for both the planning and execution of goal-directed behavior and also the focusing of attention onto currently relevant sensory input^58–60^.

In summary, based on our data, we argue that 8Av/45 receives bottom-up visual input allowing it to represent colors. Additionally, a fast and robust evaluation of that input related to current behavioral goals takes place. The current task demands determine how such a signal will be used by the animal, such that both the generation of top-down attention and also decision-making processes may equally benefit from the information available in 8Av/45.

## Materials and Methods

Research with non-human primates represents a small but indispensable component of neuroscience research. The scientists in this study are aware and are committed to the great responsibility they have in ensuring the best possible science with the least possible harm to the animals^61^.

Methodological details concerning our animal subjects, their holding and welfare, our experimental permits, surgeries and implants as well as details of the experimental setup were reported previously^38,62,63^. We reiterate relevant details here:

### Subjects and animal welfare

Two male macaque monkeys (EDG and SUN), both 13 years old, and weighing 9 kg and 13 kg participated in the experiment. Both animals were implanted with custom-made titanium head-posts before training on the behavioral task. Subsequently, each monkey was implanted with a 4 mm^2^ × 1.5 mm 96-channel ‘Utah’ micro-electrode array (Blackrock, USA) in the left lateral prefrontal cortex (details of the surgery were reported elsewhere^38^).

All animal procedures of this study were approved by the responsible regional government office (Niedersaechsisches Landesamt fuer Verbraucherschutz und Lebensmittelsicherheit (LAVES)) under the permit number 3392 42502-04-13/1100 and were performed in full accordance with relevant guidelines and regulations.

The animals were pair-housed in the facilities of the German Primate Center (DPZ) in Goettingen, Germany. The facility provides the animals with an enriched environment including a multitude of toys and wooden structures^64,65^, natural as well as artificial light and exceeding the size requirements of the European regulations, including access to outdoor space. The animals’ psychological and veterinary welfare was monitored by the DPZ’s staff veterinarians, the animal facility staff and the lab’s scientists, all specialized on working with non-human primates. We have established a comprehensive set of measures to ensure that the severity of our experimental procedures falls into the category of mild to moderate, according to the severity categorization of Annex VIII of the European Union’s directive 2010/63/EU on the protection of animals used for scientific purposes^66^. During the study the animals had unrestricted access to food and fluid, except on the days where data were collected or the animal was trained on the behavioral paradigm. On these days, the animals were allowed access to fluid through their performance in the behavioral paradigm. Here the animals received fluid rewards for every correctly performed trial.

Surgeries were performed aseptically under gas anesthesia using standard techniques, including appropriate peri-surgical analgesia and monitoring to minimize potential suffering. The two animals were healthy at the conclusion of our study and were subsequently used in other studies.

### Apparatus

Monkeys were seated in a primate chair at a viewing distance of 102 cm from a back projection screen (171.5 × 107.2 cm image size). Two projectors (Projection Design F22, Norway) were used to display stereoscopic stimuli with a 60 Hz refresh rate and a resolution of 1920 × 1200 pixels. We used two sets of linear polarizing filters to deliver separate images to the monkeys’ eyes. Visual stimuli were presented perimetrically on a virtual spherical bowl with constant binocular disparity.

Eye position was recorded binocularly with an Eyelink 1000 system (SR-Research, Canada) at a sample rate of 500 Hz. The eye position system was calibrated with a custom 3D calibration routine prior to each experimental session. To indicate a target stimulus, monkeys responded by depressing a mechanical lever and then received a liquid reward for each correct answer (both for hit trials and correct rejections). The experiment was controlled by an Apple computer (Mac Pro 2010) running the open-source software MWorks 0.5 (mworks-project.org).

### Stimuli and procedure

A fixation point central to a random dot stereogram (3 × 3 degrees, displayed at the center of the screen and at 0° disparity) instructed the monkeys to maintain fixation within a sphere with a radius of 1.2 degrees around the fixation point and to depress and release the lever to initiate an experimental trial. Trials in which monkeys broke fixation, or in which eye blinks occurred before offset of the test stimulus were immediately aborted. Upon trial start, we presented colored random dot patterns (RDP) with a dot luminance of 19 cd/deg2, a radius of 3 degrees and a dot-density of 0.5 dots/deg^2^ on a grey background (13 cd/deg^2^). The first presentation of the RDP (*sample*) occurred at 0° disparity and centered 4.7° left of the fixation point for monkey SUN. For monkey EDG, the sample position varied between recording sessions and equaled the test stimulus position. The sample was presented for 1 second, followed by a variable memory period (800–1600 ms).

The sample dots were either light grey, or had one of four isoluminant colors (orange, cyan, green, magenta) and they either moved coherently (100% coherency) in a cardinal direction or moved incoherently on individual linear paths. The precise sample composition instructed the monkeys which out of three possible task rules they had to follow and which stimulus feature to match (see also Fig. 1): either only the direction, only the color, or the presented conjunction of direction and color was relevant for subsequent target identification.

After the memory period, a second RDP (*test*) was displayed for 250 ms at a location optimal for a concurrently recorded MT single unit. While the response data of recorded MT units are not part of this report, the properties of those units (their preferred binocular disparities and receptive field locations) determined where the test stimuli were shown in each session. Throughout a recording session however, the test location stayed constant. Test RDPs were always located in the monkey’s right hemifield (Fig. 1), were always colored and always moved coherently (100% coherency) in a cardinal direction. In trials in which the sample contained a motion direction, the test moved either in this, or the opposite motion direction. Similarly, when the sample contained a color other than grey, the test was either colored with the same color, or with the ‘opposite’ color (Fig. 1C).

The monkeys were trained to indicate a matching test stimulus by depressing the response button within 600 ms of test stimulus onset. No response within this time window was counted as rejection of the test as non-match. Rewards were delivered immediately after correct responses, or after 600 ms following test onset in case of correct rejections.

The three trial types (single color, single motion, and conjunction matching) were presented pseudo-randomly interleaved throughout each session. The trial randomization procedure sampled from a trial pool in which every possible stimulus configuration within each trial type was equally likely and each test stimulus was also equally likely to be a target or non-target. Throughout a session, trials were sampled without replacement from this pool, unless the monkeys did not correctly respond to the stimulus in a given trial, or fixation was lost before responses could be recorded. In this case, the respective trial re-entered the pool and was re-tested at another time during the session, but still counted in the analysis of overall task performance. This was done to counteract possible behavioral biases the monkeys would develop for any kind of stimuli, task type or response. Similarly, high task performance was encouraged by a reward scheme that increased reward amounts for correctly answered trials in straight succession. Throughout the session, the monkey’s motivation to perform the tasks was also monitored by the experimenter and the reward was gradually increased throughout the session to keep motivation and task performances high.

### Data acquisition and preprocessing

We recorded Local Field Potentials (LFP) simultaneously from all channels of 96-channel ‘Utah’ arrays (BlackRock, USA) implanted in each monkey’s left lateral prefrontal cortex (lPFC). LFPs were amplified and then digitized at a sampling rate of 40 kHz using a Plexon Omniplex system. Overall, we recorded 16 sessions from monkey EDG and 25 sessions from monkey SUN. For monkey SUN, we completely excluded 8 recording channels that appeared broken or disconnected according to visual inspection.

For each session, we then extracted RAW recordings for all correct trials (i.e. hit trials and correct rejections), which also passed a check for signal clipping artifacts. We removed line-noise by means of a 50 Hz notch filter and low-pass filtered the signals at 150 Hz. Next, the data was resampled to 250 Hz and then band-pass filtered from 1–80 Hz. Finally, we time-locked trial segments to either the test stimulus onset or sample stimulus onset, as needed. Trials in which button presses occurred were not truncated at the response time. Those analyses were performed using Matlab R2017b and the fieldtrip toolbox 20161129^67^.

### Decoding analysis

We began each decoding analysis by selecting and pooling all trials with the desired types, separately for each monkey. If the analyzed trials contained button presses, we excluded any trials with reaction times of less than 300 ms. We then constructed balanced, 20-fold cross-validation splits, such that across all splits, each trial appeared once in a testing set and both training and testing sets were balanced according to trial types. This ensured that the chance performance of decoding for both training and testing splits was equal to the reciprocal of the number of unique trial types. Trials that were over-represented were excluded randomly.

For each time-point, trial and recording channel we then extracted pre-processed LFP voltages from a temporal window with a 2-sample radius (i.e. a maximum width of 5 samples, or 20 ms). Because this inflated the number of samples by a factor of up to 5, we next reduced the dimensionality of the training splits by averaging always 5 similar, but otherwise randomly selected trials (i.e. with the same type) in each training set. We then trained linear support vector machines (C = 1) for each training fold and time-bin and tested their accuracies on the corresponding left-out testing splits. Finally, we averaged decoding accuracies for each time-point across all testing folds.

In order to contrast the resulting average decoding accuracies with their empirical noise distributions, we randomly permuted the trial labels of the training sets after averaging across trials and before training of the classifiers. We then repeated the above analysis, including trial selection and averaging, for 500 times and tested with z-statistics whether the training on original data yielded significantly higher decoding performances than expected by chance.

Those analyses were performed on a computational cluster running Matlab R2012b, the CoSMoMVPA toolbox 1.1.0^68^ and libSVM 3.22^69^.

### Data visualization and availability

For data visualization, but not statistical testing, we calculated the moving averages in 28 ms sliding-windows for each decoding time-course. We plotted this data along corresponding 95% confidence intervals (Figs. 3–5). For Figs. 2–5 and all online Supplementary Figures we used Matlab and the gramm toolbox^70^.

We provide full access to the data and all our custom analysis scripts used to process, analyze and plot them, upon reasonable request.

## Supplementary information

Supplementary Figures and Videos.

Video S6.

Video S9.

Video S12.
