# Supplementary material for: The lateral prefrontal cortex of primates encodes stimulus colors and their behavioral relevance during a match-to-sample task
Source: Sci Rep. 2020 Mar 6;10:4216. doi: 10.1038/s41598-020-61171-3 (PMC7060344; doi:10.1038/s41598-020-61171-3)
Supplement: Supplementary file 1 — Supplementary Figures and Videos. [file 41598_2020_61171_MOESM1_ESM.pdf]

Supplementary figures and videos

The lateral prefrontal cortex of primates encodes stimulus colors and their behavioral relevance during a match-to-sample task

Philipp Schwedhelm, Daniel Baldauf, Stefan Treue

Figure S1

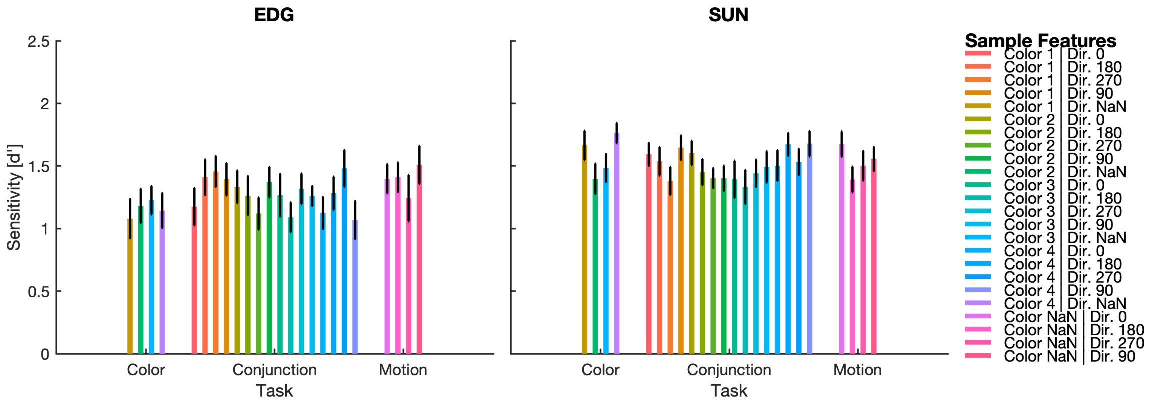

Task performances were calculated separately for each sample identity. Sensitivity indices d' were calculated separately for sample type and then plotted according to task type (like Fig. 2, main manuscript). Error bars represent standard errors across n=16 and n=25 sessions, for monkey EDG and SUN, respectively.

Figure S2

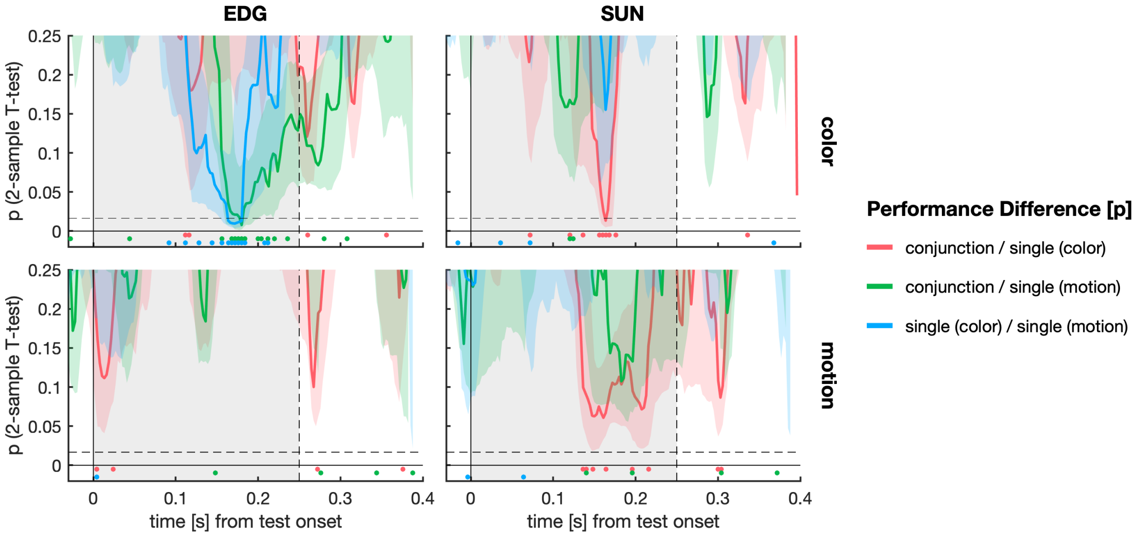

The decoding of test color and test motion was not statistically different between task types. We tested with two-sample t-tests whether the decoding performances across groups of N=20 testing folds were different

between task types. We evaluated the significance of these tests based on Bonferroni corrected α=0.0167, for each time-point. Dots in corresponding colors above the x-axes indicate for which time-bins the difference between two conditititions was significant. Because of the multiple comparisons, we defined epochs of significant differences to be at least 40ms wide and contain only significant differences between conditions. This procedure was used before for estimation of decoding onsets (see Fig. 3-5 main manuscript and text; Fig. S3). Here, we found that for no comparison between two conditions, no monkey (columns left and right) and no feature (upper row: color, lower row: motion) such significant epochs existed.
The grey, shaded area illustrates the test stimulus duration and the horizonal, dashed line indicates α=0.0167. P-values are plotted as moving averages of 28ms sliding-windows with corresponding 95% confidence intervals.

Figure S3

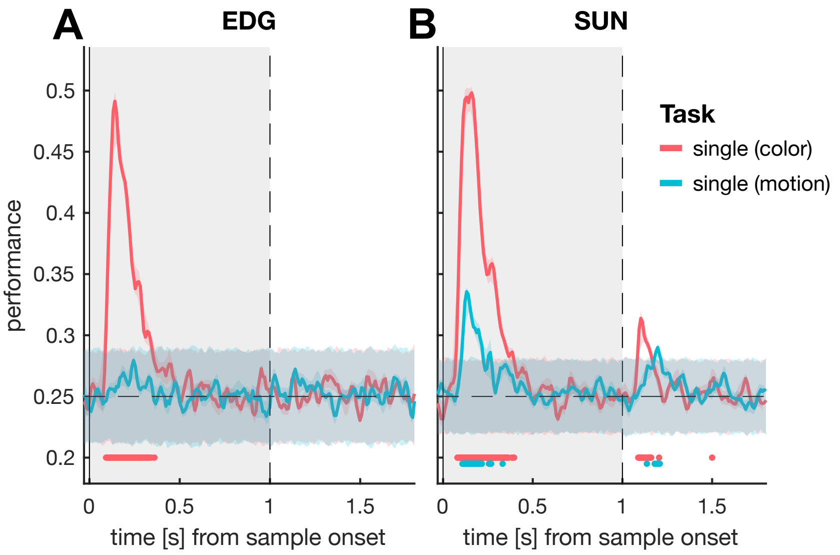

Stimulus color can be decoded from 8Av/45 activity even during sample epochs. **(A)** Similar to the test stimulus, the sample stimulus for single tasks contained the relevant feature (i.e. one out of four colors, or one out of four motion directions). The irrelevant feature dimension (i.e. motion in color trials and color in motion trials) was replaced by noise (i.e. random motion and grey color, respectively; see also Fig. 1, main manuscript). Here we trained support vector machines to separate four relevant stimulus features based on time-locked 8Av/45 data. We used the same procedure as for the test stimuli (see Fig. 3-5, main manuscript), but calculated statistics based on only 100 shuffle runs. In short, the y-axis indicates decoding performance based on a 20-fold cross-validation procedure and the x-axis indicates time locked to sample stimulus onset. The sample was presented for 1s (grey, shaded area), followed by a variable memory period. Above the x-axis, dots in corresponding colors indicate for which time-bins the decoding probability significantly differs from chance, evaluated at a significance level of α=1e-4. The time of onset of decoding was estimated by determining the mid of the first 40ms epoch that contained only significant samples. For monkey EDG, motion decoding from motion task samples did not reach significance, but color decoding from color task samples became significant at 112ms after sample stimulus onset. **(B)** Like A, but for monkey SUN. Here, sample motion could be decoded 128ms after stimulus onset, and color could be decoded starting 100ms after stimulus onset. **(A/B)** Performances were plotted as moving averages of 28ms sliding-windows with corresponding 95% confidence intervals. Chance performances were plotted as 99.9% confidence intervals of the shuffle distributions as overlapping, shaded areas.

Figure S4

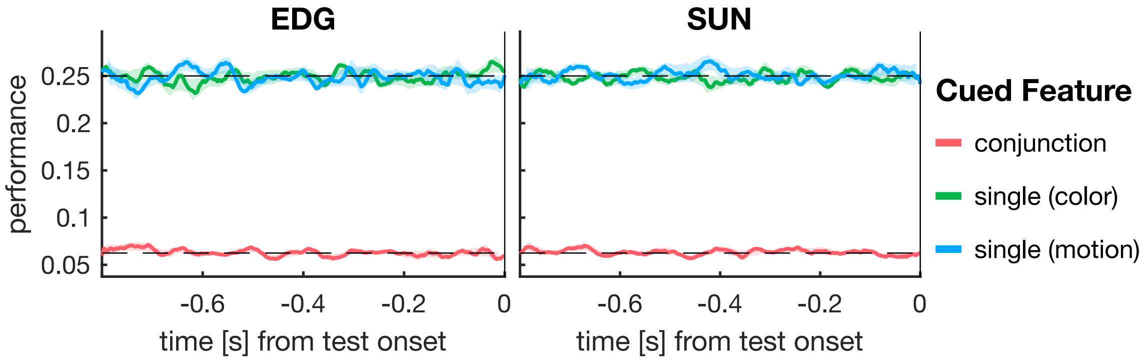

No recall of sample information before stimulus onset. We trained support vector machines to separate individual trials based on their previously presented sample features. For both monkeys, we find that during 800ms before test stimulus onset (variable memory period), the memorized features cannot be (linearly) read out from time-locked 8Av/45 data. Here we used the same analysis pipeline as for the test and sample stimuli (see Figures 3-5, S3), but did not calculate statistics due to the fact that all decoding performances in all time-bins fluctuated tightly around their corresponding chance performances (dashed lines). For conjunction trials, the chance performance was p=1/16 (4 motion directions x 4 colors, see also Fig. S1). Decoding performances were plotted as moving averages of 28ms sliding-windows with corresponding 95% confidence intervals.

Figure S5

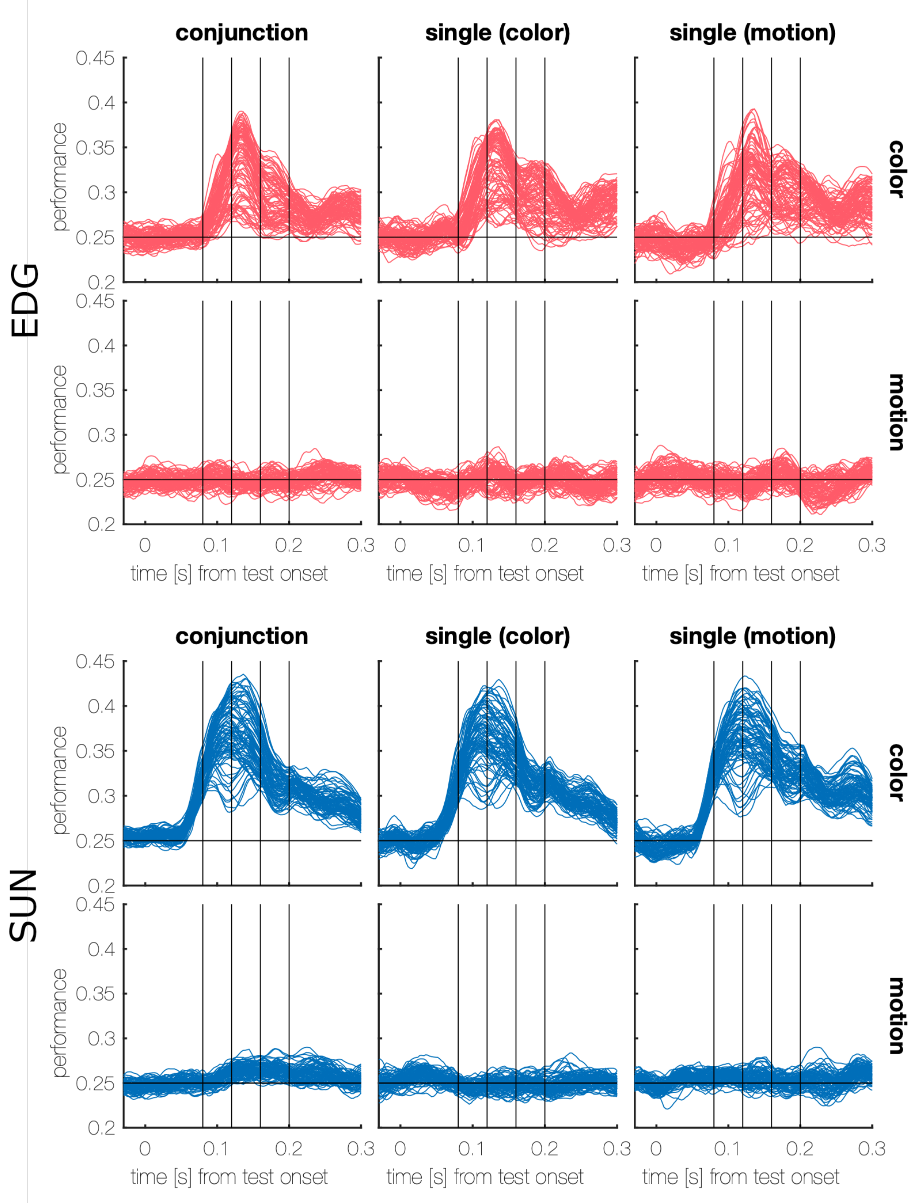

Stimulus color can be decoded from single sites of 8Av/45 electrode arrays. We trained support vector machines separately for every site (electrode) of the prefrontal arrays, attempting to decode the test stimulus color and motion in all three task types. For both monkeys, we find that similar to the combined analysis of all electrodes (see main manuscript, Fig. 3), single electrode recordings were informative about stimulus color, but not motion direction. Black vertical lines indicate time-points at 80ms, 120ms, 160ms and 200ms following test stimulus onset and match to indicated time-points in corresponding video S6.

Video S6

Specific clusters of electrodes best encode stimulus color. We projected the data shown in Fig. S5 back to the electrode arrays' spatial outlines and then animated the progression of decoding over time. The video pauses briefly at the time-points indicated in Fig. S5.

Video file available online.

Figure S7

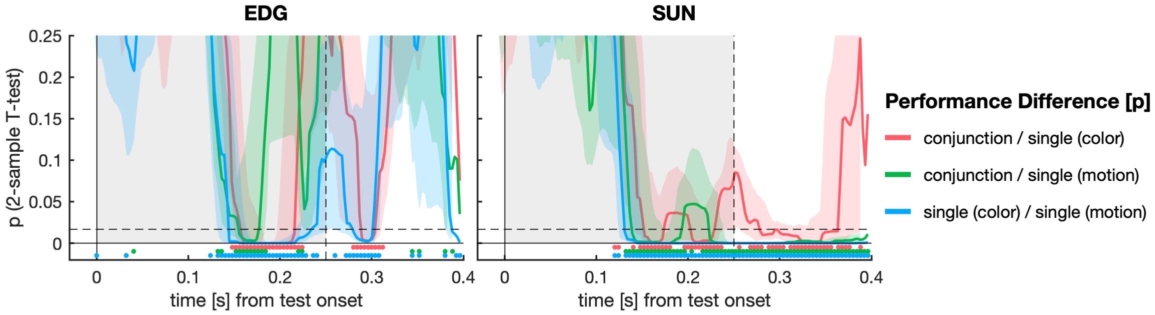

The decoding of trial-by-trial behavior was statistically different between task types. We tested with two-sample t-tests whether the decoding performances across groups of N=20 testing folds were different between task types. We evaluated the significance of these tests based on Bonferroni corrected α=0.0167, for each time-point. Dots in corresponding colors above the x-axes indicate for which time-bins the difference between two condititions was significant. Because of the multiple comparisons, we defined epochs of significant differences to be at least 40ms wide and contain only significant differences between conditions, as was done before for analyses plotted in Figs. 3-5 main manuscript and Figs. S2-S3). Here we found that between task conditions, decoding performances became significantly different between color and conjunction trials after 168ms for EDG and 216ms for SUN. Color and motion trials were significantly different already after 152ms for both monkeys. Between motion and conjunction trials, the difference reached significance after 152ms for monkey SUN, but did not become significant for monkey EDG.
The grey, shaded area illustrates the test stimulus duration and the horizonal, dashed line indicates α=0.0167. P-values are plotted as moving averages of 28ms sliding-windows with corresponding 95% confidence intervals.

Figure S8

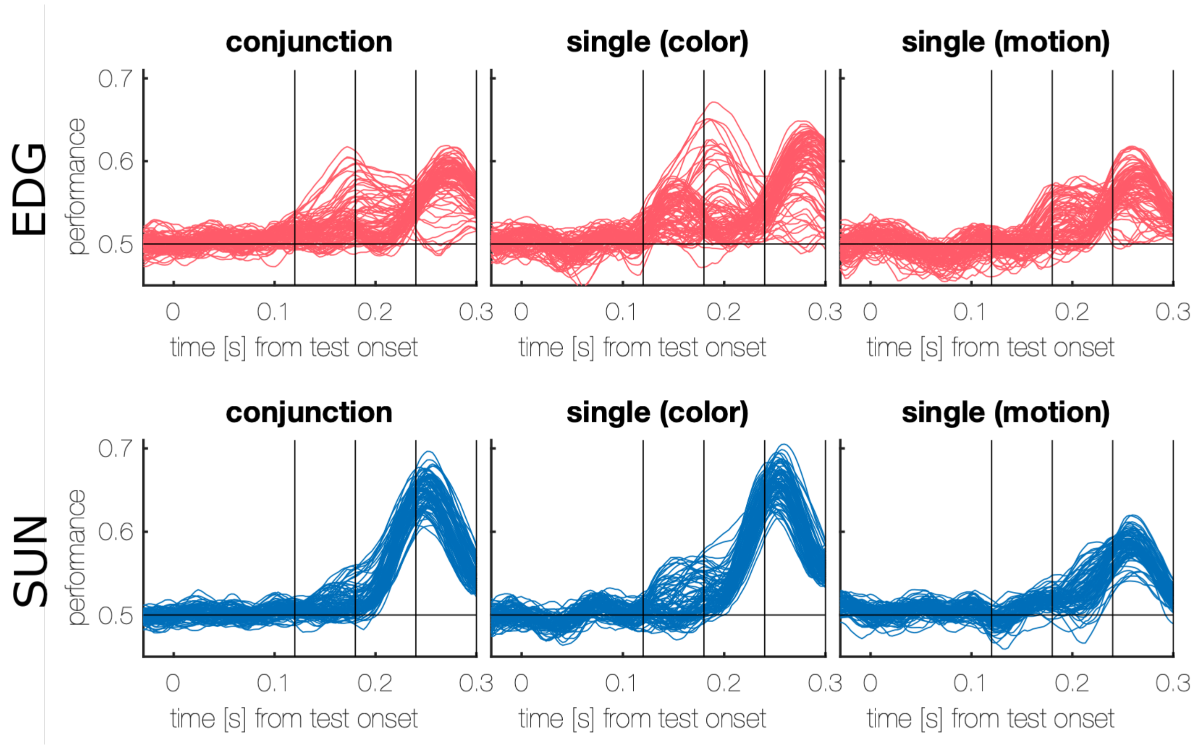

Trial-by-trial behavior can be predicted from single electrode sites, especially in monkey EDG. We trained support vector machines separately for every site (electrode) of the prefrontal arrays, attempting to predict the monkeys' behavior. Especially for monkey EDG and during color task trials (see also main manuscript, Fig. 4), single electrode recordings were informative about trial-by-trial behavior. Black vertical lines indicate time-points at 120ms, 180ms, 240ms and 300ms following test stimulus onset and match to indicated time-points in corresponding video S9.

Video S9

Specific clusters of electrodes best predict trial-by-trial behavior. We projected the data shown in Fig. S8 back to the electrode arrays' spatial outlines and then animated the progression of decoding over time. The video pauses briefly at the time-points indicated in Fig. S8. Note the distinct clusters of electrodes with high decoding performance around t=180ms.

Video file available online.

<u>Figure S10</u>

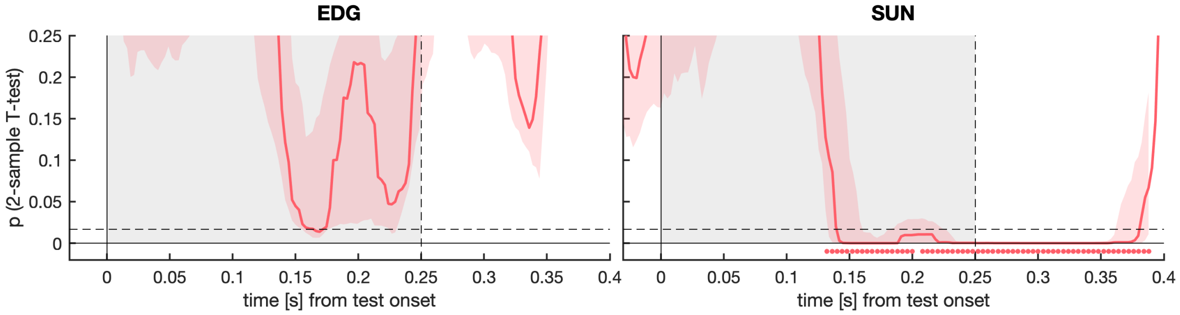

The decoding of color-match and motion-match information was statistically different for monkey SUN but not for monkey EDG. We defined epochs of significant differences to be at least 40ms wide and contain only significant differences between conditions, as was done before for all similar analyses plotted in Figs. 3-5 main manuscript and Figs. S2-S3, S7). Here, the difference between color matching and motion matching performance does not become significant for monkey EDG. For monkey SUN, color and motion match performances are significantly different starting at 152ms.

Dots in corresponding colors above the x-axes indicate for which time-bins the difference between motion- and color-match decoding performance was significant. Grey, shaded areas illustrate test stimulus duration and the horizonal, dashed line indicates α=0.0167. P-values are plotted as moving averages of 28ms sliding-windows with corresponding 95% confidence intervals.

Figure S11

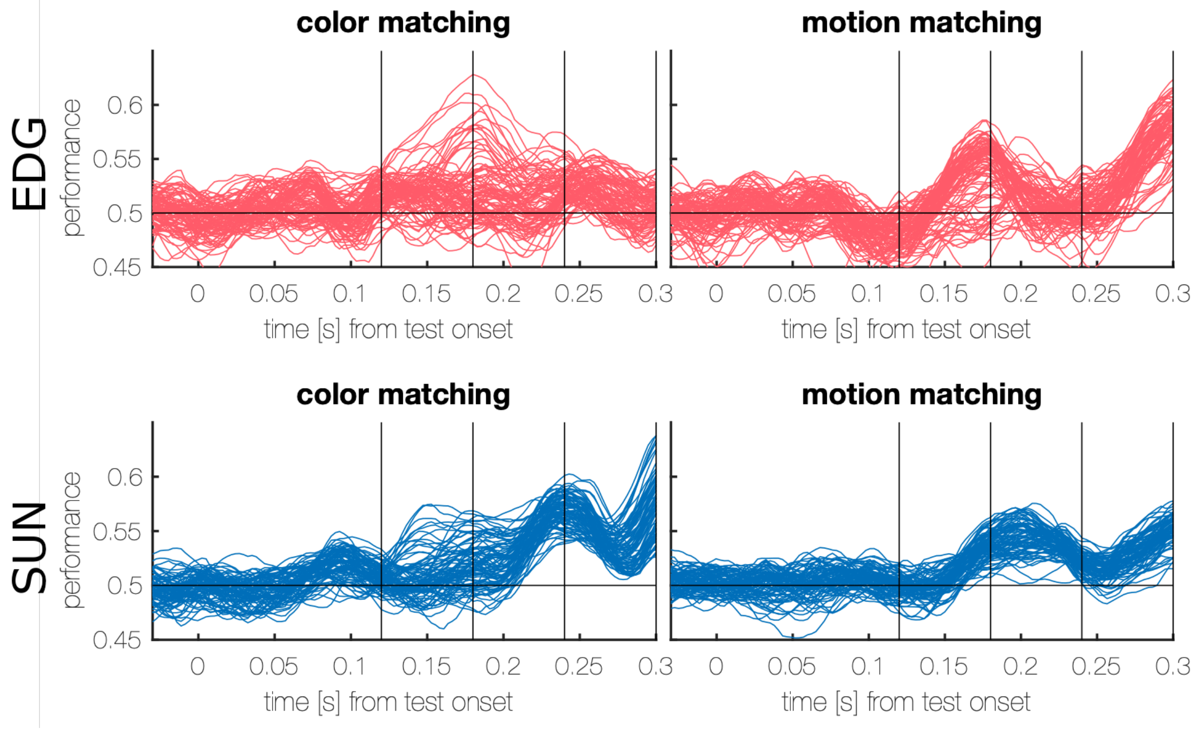

Color-match information decoded based on single channel LFPs. We trained support vector machines separately for every site (electrode) of the prefrontal arrays, attempting to decode matches in the color or motion feature during the combined task (equivalent to the analysis underlying main manuscript Fig. 5). Black vertical lines indicate time-points at 120ms, 180ms, 240ms and 300ms following test stimulus onset and match to indicated time-points in corresponding video S12.

Video S12

Specific clusters of electrodes best predict color matches. We projected the data shown in Fig. S11 back to the electrode arrays' spatial outlines and then animated the progression of decoding over time. The video pauses briefly at the time-points indicated in Fig. S11. Note the distinct clusters of electrodes with high decoding performance around t=180ms. Note the similarity of these clusters to the groups of electrodes best predicting trial-outcomes at the same time-point (Fig. S8 and video S9).

Video file available online.
